# Supplementary material for: Cannabis use and the risk of primary open-angle glaucoma: a Mendelian randomization study
Source: Sci Rep. 2023 Nov 10;13:19605. doi: 10.1038/s41598-023-45872-z (PMC10638381; doi:10.1038/s41598-023-45872-z)
Supplement: Supplementary file 1 — Supplementary Information. [file 41598_2023_45872_MOESM1_ESM.pdf]

Cannabis use and the risk of primary open-angle glaucoma: a two-sample Mendelian randomization study

Authors: Andreas Katsimpris, Sebastian-Edgar Baumeister, Hansjörg Baurecht, Andrew J Tatham, Michael Nolde

Supplementary Figures and Tables

Supplementary Figure S1 Funnel plot of single SNP Wald ratio estimates for the effect of lifetime cannabis use on primary open-angle glaucoma

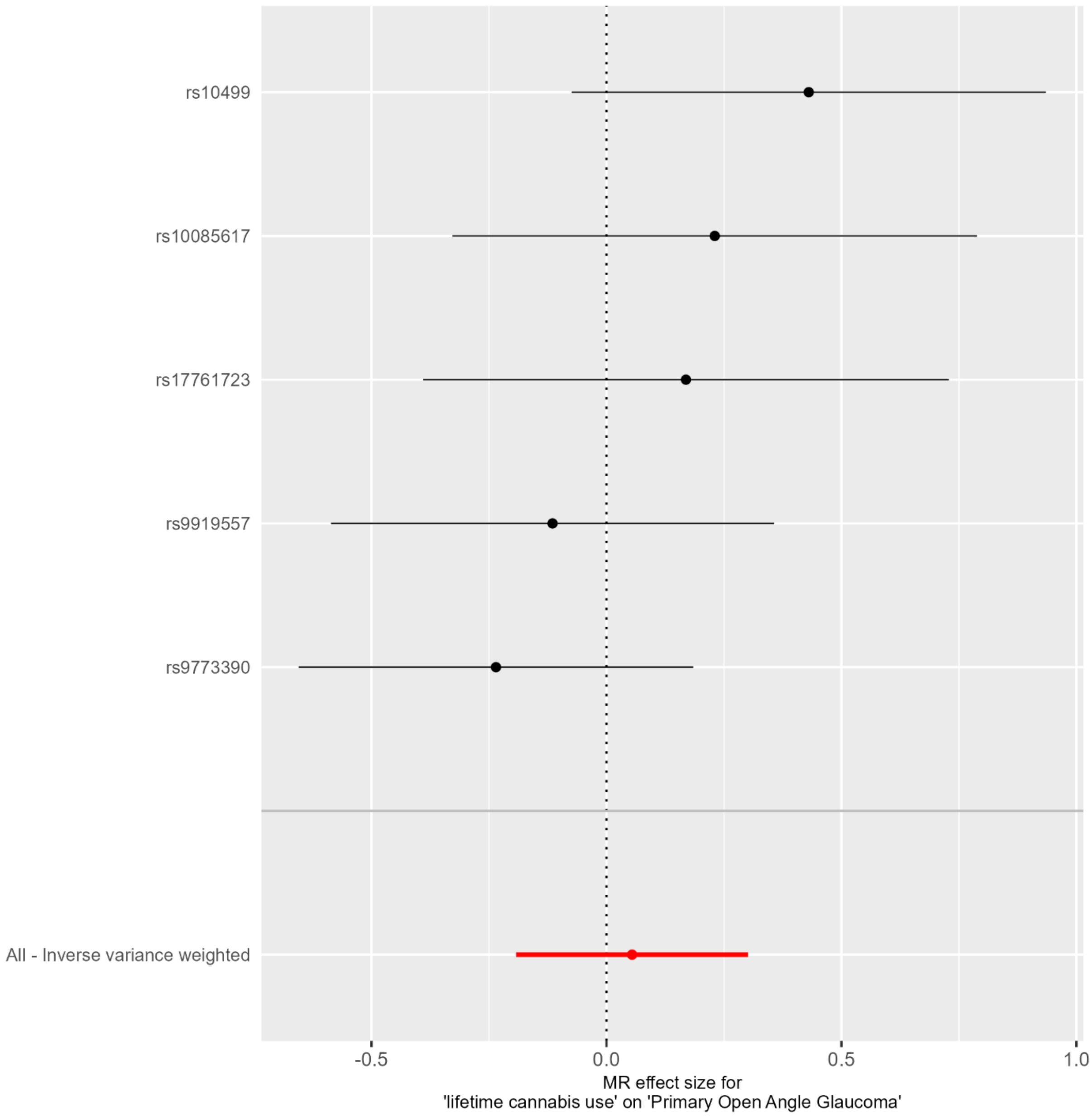

SNP: single-nucleotide polymorphism

Supplementary Figure S2 Funnel plot of single SNP Wald ratio estimates for the effect of cannabis use disorder on primary open-angle glaucoma

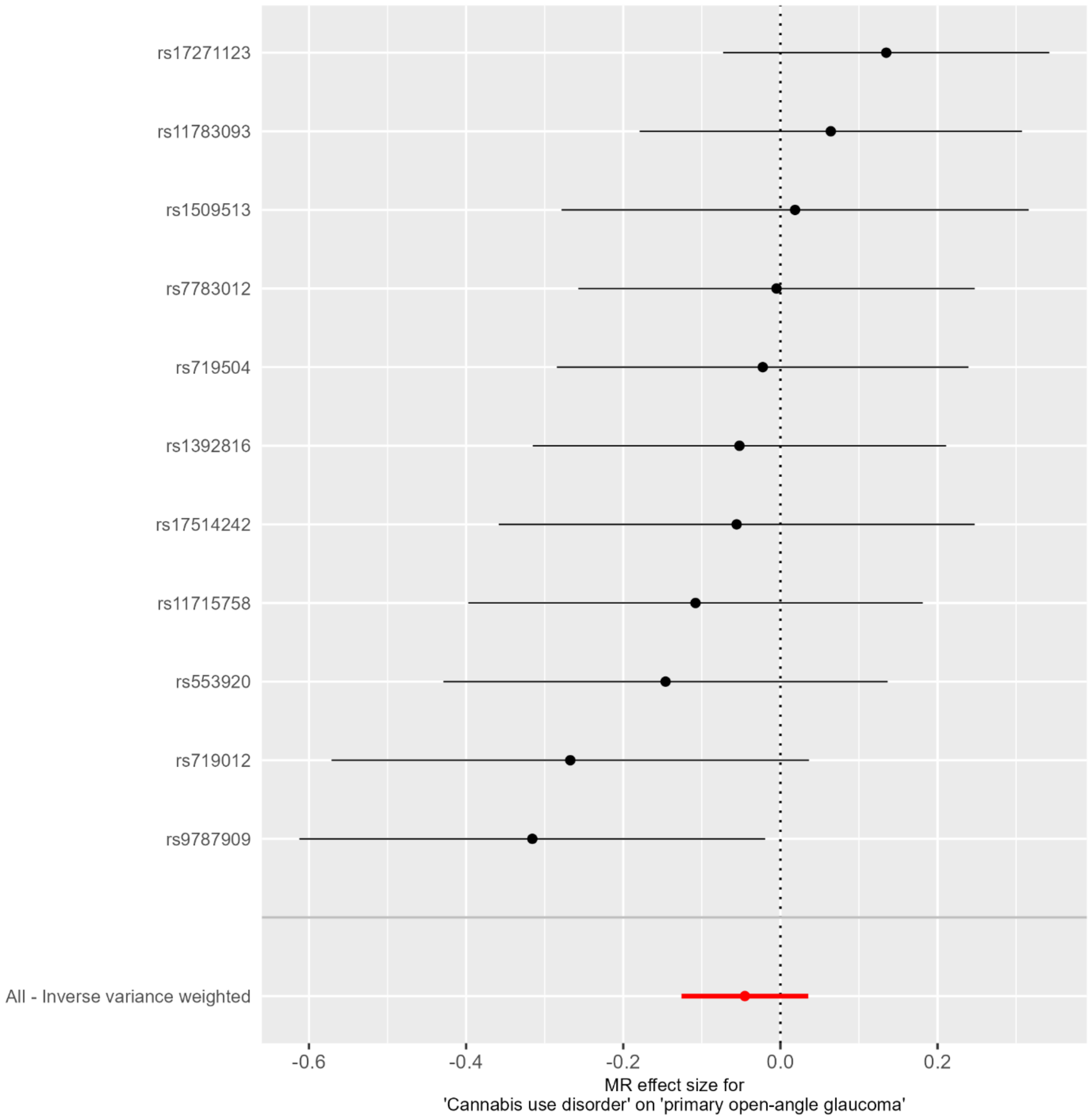

SNP, single-nucleotide polymorphism

Supplementary Figure S3 Scatter plot of SNP-primary open-angle glaucoma associations vs SNP-lifetime cannabis use associations

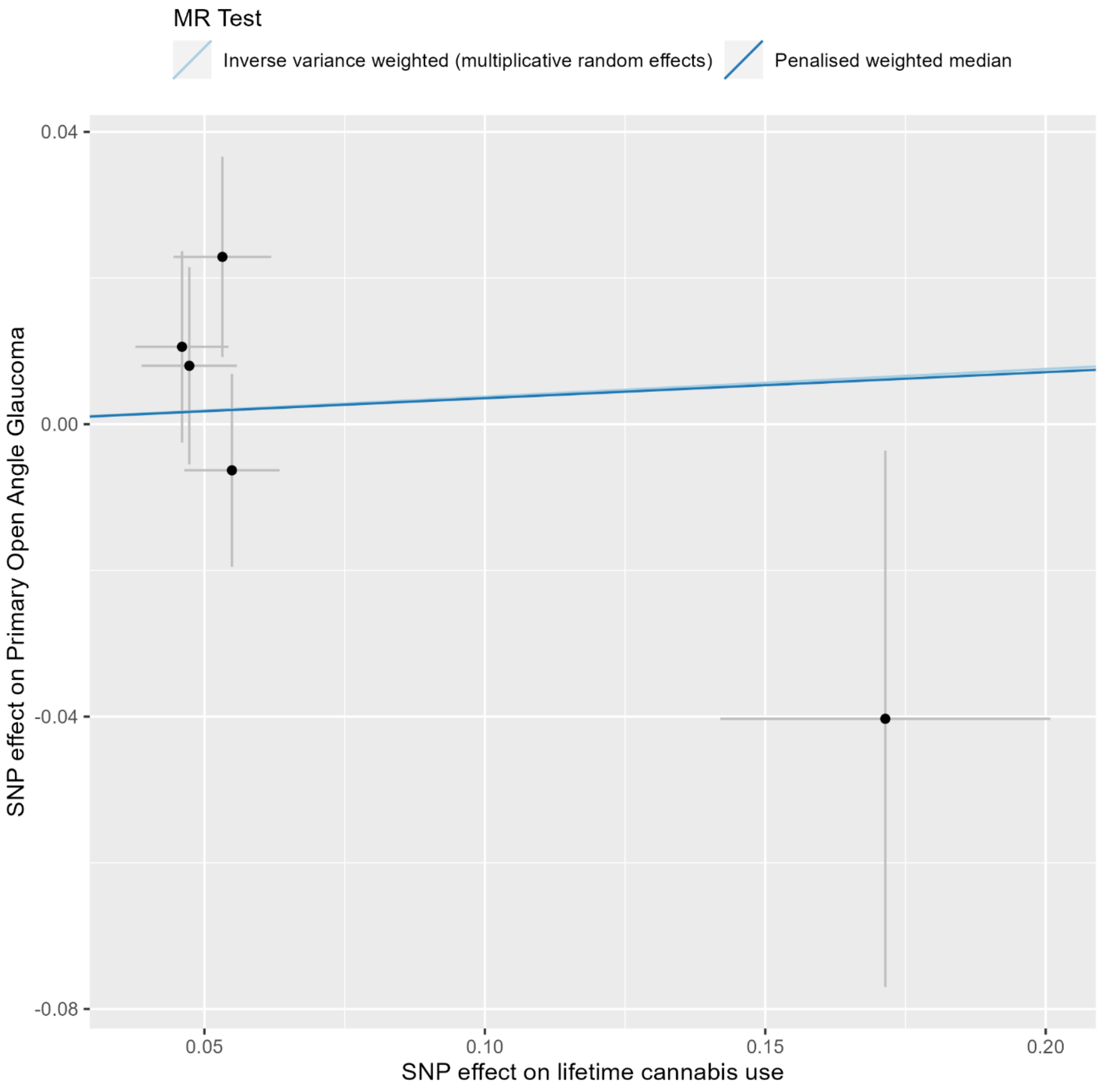

SNP: single-nucleotide polymorphism

Supplementary Figure S4 Scatter plot of SNP-primary open-angle glaucoma associations vs SNP-cannabis use disorder associations

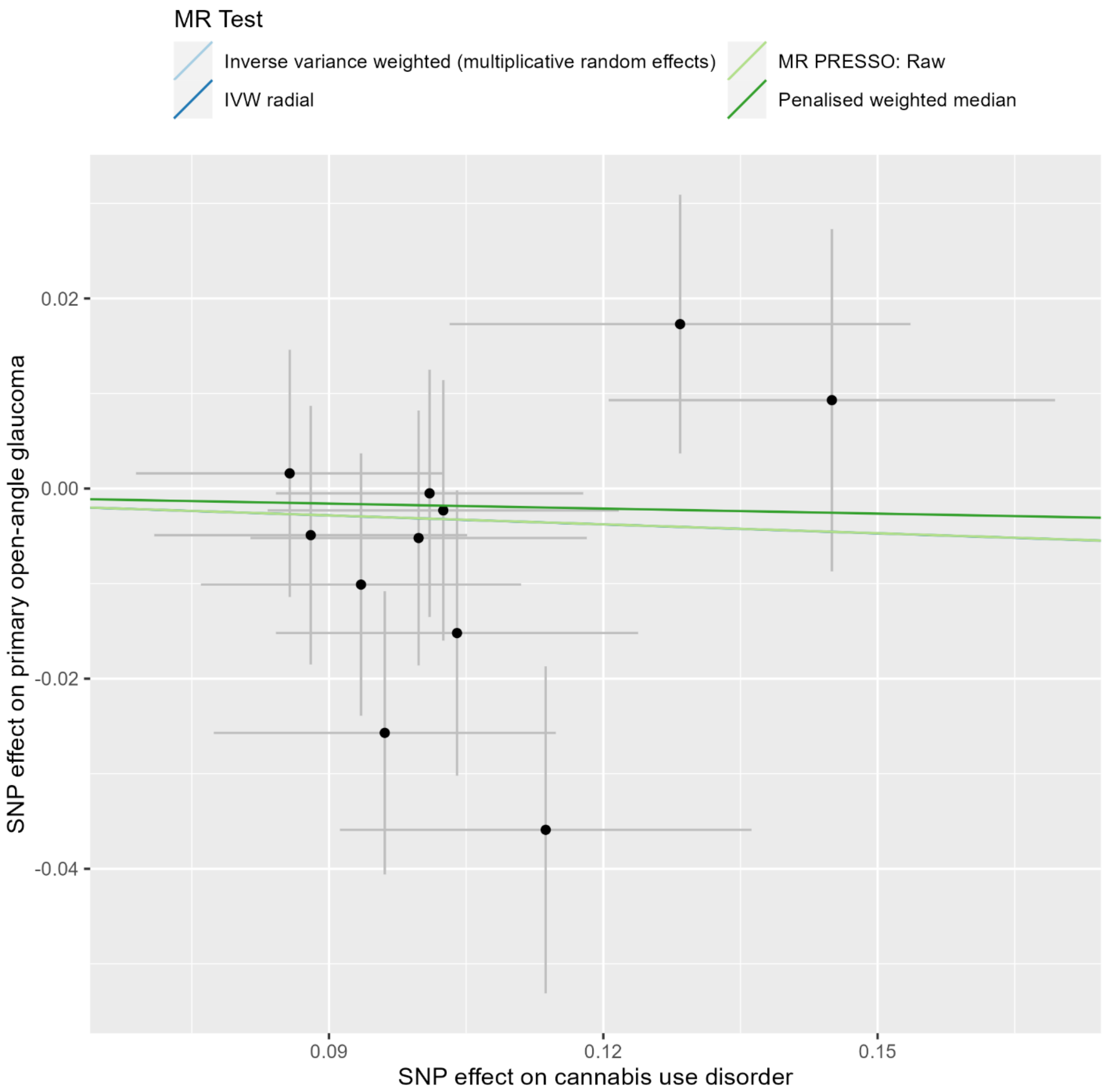

SNP: single-nucleotide polymorphism

Supplementary Table S1 Phenotypic descriptive statistics of studies included in the exposure, confounder/outcome risk factor, and outcome genome-wide association studies

GWAS / Study	Phenotype			
(Gharahkhani et al., 2021)	Primary open angle glaucoma			
	N cases	N controls	Age (mean ± SD) cases	Age (mean ± SD) controls
NEIGHBOR/MEEI	2606	2606	65.7 ± 13.4	68.3 ± 11.4
EPIC-Norfolk Eye Study	664	5630	61.5 ± 7.9	55.6 ± 7.9
ANZRAG	3071	6750	NA	NA
UKBB POAG ICD10 code	1448	22107	62.67 ± 5.6	56.36 ± 7.9
Kaiser Permanente GERA Cohort	3819	47961	79.6 ± 9.4	69.7 ± 13.0
KCL	576	287	70.5±13.5	80.3±4.3
BMES	107	600	68.9 ± 7.9	63.8 ± 8.3
Southampton	941	1557	>40	>40
GHS	47	2731	62.21 ±10.63	55.66 ±10.86
HPFS illumina	116	527	58.3 ± 8.4	51.5 ± 8.0
HPFS Affy	34	1297	58.7 ± 8.0	52.8 ± 7.9
NHS illumina	266	1692	55.8 ± 6.4	53.2 ± 6.5
NHS Affy	46	1992	55.7 ± 6.0	53.0 ± 6.7
ERF	110	1999	NA	NA
Rotterdam Study I	198	1282	NA	NA
FINNGEN	1824	93036	65.6 ± 11.9	57.8 ± 16.1
Geisinger 60K	664	5904	>18	>18
Geisinger 30K	140	1622	>18	>18
(Pasman et al., 2018)	Lifetime cannabis use			
	N	Female (%)	Age (Mean)	Cannabis users (%)
ALSPAC	2,976	55.7	17.75	41.6
BLTS	721	57.1	26.2	59.5
CADD	853	30.2	24.9	79.3
EGCUT1	2,765	54.5	33.8	1.3
EGCUT2	970	51.2	31.1	4.8
FinnTwin	1,029	51.7	22.8	27.4

HUVH	981	29.8	35.6	20.1
MCTFR	6,241	53.5	37.2	59.3
NTR	4,653	66.2	36.9	26.9
QIMR	6,778	53.8	45.2	51.4
TRAILS	1,226	46.9	19.0	50.5
Utrecht	1,173	53.5	20.6	54.1
Yale Penn EA	1,964	40.2	38.2	91.6
Radar	338	44.4	19.54	58.8
SYS	551	56	49.52	52.45
TwinsUK	2,070	92.6	57.7	11.7
Yale Penn AA	2,660	46.4	41.6	81.8
23andme	22,683	55.3	54	43.2
UKB	126,785	56.3	55	22.3
(Johnson et al., 2020)	Cannabis use disorder			
	Cases	Controls		
CATS	958	453		
CADD	397	699		
CHDS	201	420		
FSCD	226	314		
COGEND Nico	306	607		
COGEND SAGE	228	830		
GEDI-GSMS	81	491		
BLTS	170	1216		
MCTFR	449	1625		
Yale Penn 1	916	833		
Yale Penn 2	557	497		
bigCOGA	2206	5053		
CEDAR	64	148		
OZ-ALC	593	4893		
VTSABD	99	734		
IASPSAD	104	613		

Add health	722	4071	
iPSYCH	2758	53326	
deCODE	6033	280396	
(Pulit et al., 2019)	Body mass index		
	N	BMI (Mean ± SD)	Female (%)
UKB + GIANT	806,834	27.4 (4.8)	54

Supplementary Table S2 Associations of single nucleotide polymorphisms for lifetime cannabis use and cannabis use disorder

			Estimates for exposure					Estimates for primary open angle glaucoma		
SNP	EA	OA	EAF	BETA	SE	P	F	BETA	SE	P
Lifetime cannabis use										
rs10085617	A	T	0.416	0.046	0.008	2.9e-08	30.7	0.011	0.013	0.209
rs10499	A	G	0.651	0.053	0.009	1.1e-09	37.4	0.023	0.014	0.047
rs17761723	T	C	0.346	0.047	0.009	3.2e-08	31.0	0.008	0.014	0.277
rs9773390	T	C	0.933	-0.171	0.029	5.7e-09	34.0	0.040	0.037	0.136
rs9919557	T	C	0.614	-0.055	0.009	9.9e-11	41.7	0.006	0.013	0.317
Cannabis use disorder										
rs11715758	A	G	0.622	-0.094	0.018	8.9e-08	28.5	0.010	0.014	0.232
rs11783093	T	C	0.846	-0.145	0.024	2.7e-09	35.3	-0.009	0.018	0.303
rs1392816	T	C	0.624	-0.100	0.018	6.1e-08	29.4	0.005	0.013	0.349
rs1509513	A	G	0.546	0.086	0.017	3.2e-07	26.0	0.002	0.013	0.451
rs17271123	T	G	0.587	0.128	0.025	3.5e-07	26.0	0.017	0.014	0.102
rs17514242	C	G	0.649	-0.088	0.017	2.6e-07	26.5	0.005	0.014	0.359
rs553920	T	C	0.770	0.104	0.020	1.6e-07	27.6	-0.015	0.015	0.155
rs719012	T	C	0.264	0.096	0.019	2.9e-07	26.4	-0.026	0.015	0.042
rs719504	A	G	0.650	0.102	0.019	9.0e-08	28.5	-0.002	0.014	0.433
rs7783012	A	G	0.476	0.101	0.017	1.8e-09	36.1	0.000	0.013	0.485
rs9787909	A	C	0.829	0.114	0.022	4.5e-07	25.5	-0.036	0.017	0.018

EA, effect allele. OA, other allele. EAF, effect allele frequency. SE, standard error.

Supplementary Table S3 Association ($P<5x10^{-8}$) of the single nucleotide polymorphisms used as instruments with confounders or outcome risk factors in PhenoScanner (accessed on 2022/11/20 using the phenoscanner function of the R MendelianRandomization package)

SNP	Phenotypes	PMID
Lifetime cannabis use		
rs10499	Mean corpuscular volume	27863252
rs10499	Red blood cell count	27863252
rs10499	Hip circumference	25673412
rs10499	Waist circumference	25673412
rs10499	Crohns disease	26192919
Cannabis use disorder		
rs11715758	High light scatter percentage of red cells	27863252
rs11715758	High light scatter reticulocyte count	27863252
rs11715758	Immature fraction of reticulocytes	27863252
rs11715758	Reticulocyte count	27863252
rs11715758	Reticulocyte fraction of red cells	27863252
rs7783012	Years of educational attainment	27225129
rs7783012	Age first birth	27798627

PMID, PubMed ID. Body mass index was considered as a relevant confounder or risk factor for primary open angle glaucoma.

Supplementary Table S4 Heterogeneity of Wald ratios and MR-Egger test for directional pleiotropy

Lifetime cannabis use	Heterogeneity			
	Q	Degrees of Freedom	P	I_{GX}^2
Lifetime cannabis use	2.8	4	0.592	0.1
Cannabis use disorder	9.9	10	0.45	0.036
	MR-Egger test for directional pleiotropy			
	Intercept	Standard error	P	
Lifetime cannabis use	2.927e-02	0.018	0.197	
Cannabis use disorder	-3.637e-02	0.029	0.239	

Supplementary Table S5 Inverse variance weighted estimates in leave-one-out analysis in primary analysis

SNP excluded	SNP	OR	(95% CI)	P value
Lifetime cannabis use	rs10085617	1.02	(0.83;1.25)	0.885
	rs10499	0.98	(0.84;1.14)	0.757
	rs17761723	1.02	(0.83;1.26)	0.830
	rs9773390	1.12	(0.95;1.32)	0.169
	rs9919557	1.07	(0.87;1.32)	0.507
Cannabis use disorder	rs11715758	0.97	(0.92;1.03)	0.369
	rs11783093	0.96	(0.9;1.02)	0.179
	rs1392816	0.97	(0.91;1.03)	0.326
	rs1509513	0.97	(0.91;1.03)	0.257
	rs17271123	0.95	(0.9;1)	0.042
	rs17514242	0.97	(0.91;1.03)	0.321
	rs553920	0.98	(0.92;1.03)	0.408
	rs719012	0.98	(0.93;1.03)	0.472
	rs719504	0.97	(0.91;1.03)	0.293
	rs7783012	0.97	(0.91;1.03)	0.272
	rs9787909	0.98	(0.94;1.03)	0.514

References

Gharahkhani, P., Jorgenson, E., Hysi, P., Khawaja, A. P., Pendergrass, S., Han, X., . . . FinnGen, s. (2021). Genome-wide meta-analysis identifies 127 open-angle glaucoma loci with consistent effect across ancestries. *Nature Communications, 12*(1), 1258. doi:10.1038/s41467-020-20851-4

Johnson, E. C., Demontis, D., Thorgeirsson, T. E., Walters, R. K., Polimanti, R., Hatoum, A. S., . . . Agrawal, A. (2020). A large-scale genome-wide association study meta-analysis of cannabis use disorder. *Lancet Psychiatry, 7*(12), 1032-1045. doi:10.1016/s2215-0366(20)30339-4

Pasman, J. A., Verweij, K. J. H., Gerring, Z., Stringer, S., Sanchez-Roige, S., Treur, J. L., . . . International Cannabis, C. (2018). GWAS of lifetime cannabis use reveals new risk loci, genetic overlap with psychiatric traits, and a causal effect of schizophrenia liability. *Nature Neuroscience, 21*(9), 1161-1170. doi:10.1038/s41593-018-0206-1

Pulit, S. L., Stoneman, C., Morris, A. P., Wood, A. R., Glastonbury, C. A., Tyrrell, J., . . . Lindgren, C. M. (2019). Meta-analysis of genome-wide association studies for body fat distribution in 694 649 individuals of European ancestry. *Human Molecular Genetics, 28*(1), 166-174. doi:10.1093/hmg/ddy327
